# Double pigtail tube drainage for large multiloculated pyogenic liver abscesses

**DOI:** 10.3389/fsurg.2022.1106348

**Published:** 2023-01-12

**Authors:** Cui JinHua, Liu YaMan, Li Jian

**Affiliations:** ^1^ Department of Hepatobiliary Surgery, Affiliated Hospital of Chengde Medical College, Chengde, China; ^2^ Department of Gynaecology, Affiliated Hospital of Chengde Medical College, Chengde, China

**Keywords:** pyogenic liver abscess, multiloculated pyogenic liver abscess, double pigtail tube drainage, percutaneous needle aspiration, percutaneous catheter drainage

## Abstract

**Background:**

This study aims to investigate the efficacy and safety of double pigtail tube drainage compared with single pigtail tube drainage for the treatment of multiloculated pyogenic liver abscesses greater than 5 cm.

**Patients and Methods:**

This study retrospectively analyzed patients with pyogenic liver abscess admitted in the Affiliated Hospital of Chengde Medical College between May 2013 and May 2021. Patients with pyogenic liver abscess more than 5 cm in size, who underwent drainage of the abscess with either double pigtail or single pigtail tube, were included.

**Results:**

A total of 97 patients with pyogenic liver abscesses larger than 5 cm were studied. These included 34 patients with double pigtail tube drainage and 63 patients with single pigtail tube drainage. The postoperative hospital stay (13.39 ± 4.21 days vs. 15.67 ± 7.50 days; *P* = 0.045), and time for removal of the catheter (17.23 ± 3.70 days vs. 24.11 ± 5.83 days; *P* = 0.038) were lower in the double pigtail tube group compared with the single pigtail tube group. The rate of reduction, in three days, of c-reactive protein levels was 26.61 ± 14.11 mg/L/day in the double pigtail tube group vs. 20.06 ± 11.74 mg/L/day in the single pigtail tube group (*P* = 0.025). The diameter of the abscess cavity at discharge was 3.1 ± 0.07 cm in the double pigtail tube group as compared with 3.7 ± 0.6 cm in the single pigtail tube group (*P* = 0.047). There was no bleeding in any of the patients despite abnormal coagulation profiles. There was no recurrence of abscess within six months of discharge and no death in the double pigtail tube group. Conclusion: Double pigtail tube drainage treatment in multiloculated pyogenic liver abscesses greater than 5 cm in size, is safe and effective.

## Introduction

Pyogenic liver abscess (PLA) is a suppurative infection of the liver parenchyma. The incidence of PLA is high in Asian countries, The annual incidence of PLA in Taiwan was reported to increase from 11.15/100,000 population in 1996 to 17.59/100,000 population in 2004, showing an increasing trend (1). PLA is associated with significant in-hospital mortality which has ranged from 3%–20% in various reports (2–4). In recent decades, early diagnosis, effective antibiotic therapy and adequate drainage of pus have resulted in lower mortality, and the mortality rates having dropped to between 0.9 to 5.6% (5, 6).

Due to advances in imaging technology, percutaneous drainage has replaced open surgical drainage and is now the first-line treatment for PLA (7–9). No differences have been found in success rates with the use of percutaneous drainage in PLA whatever the location of the abscess or the size (10). Kulhari and Mandia (11) reported that percutaneous catheter drainage (PCD) was a better therapeutic option compared with percutaneous needle aspiration (PNA), especially in the case of large liquefied abscesses. They reported that patients treated with PNA improved slowly, mainly due to the viscosity of the pus and its rapid re-accumulation in the abscess cavity. For larger PLA, a single drainage tube offered a better and faster drainage of pus and resulted in a reduction in the size of the abscess cavity (10, 12). Typically, percutaneous drainage is achieved by placing a single catheter in the dependent portion of an abscess. With a single catheter, complex abscesses with multiloculation, necrotic debris, and clots, can be challenging to adequately drain and resolve. A single catheter may not be adequate for drainage of such abscesses. In this study, we explored the safety and efficacy of double pigtail (DP) tube drainage in multiloculated PLA, more than 5 cm in size.

## Patients and methods

This was a retrospective study conducted at the Affiliated Hospital of Chengde Medical College. The study protocol was approved by the Institutional Ethics Committee. The need for informed consent was waived because of the retrospective nature of the study.

**Figure 1:**
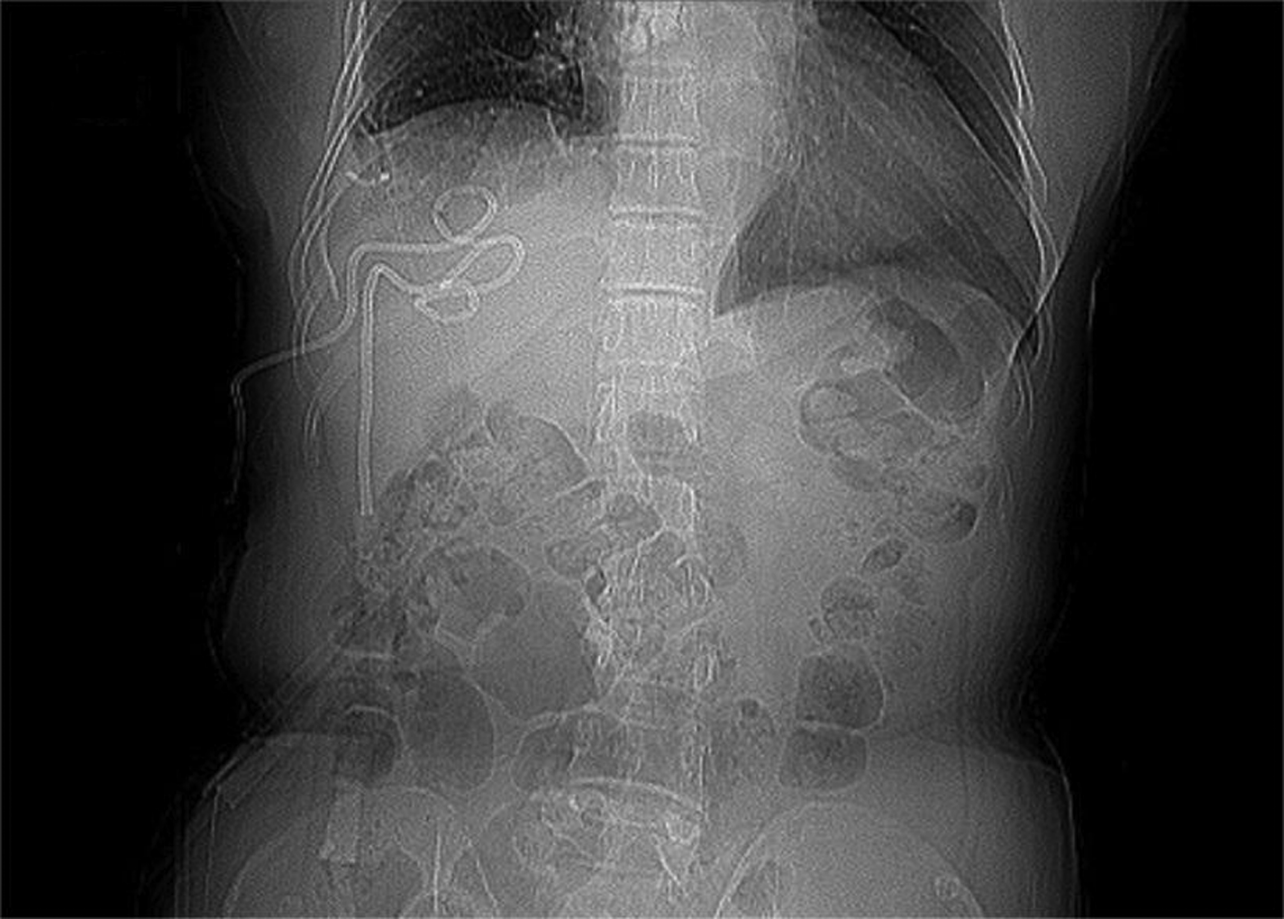
Preoperative abdominal CT suggested a large multiloculated pyogenic liver abscesses.

The medical records of all patients admitted with a diagnosis of PLA, between May 2013 to May 2021 were reviewed. Inclusion criteria included: multiloculated abscess more than 5 cm in size on computerized tomography (CT) scan or magnetic resonance imaging (MRI) of the abdomen (Figure 1); aspiration of pus on ultrasound-guided percutaneous aspiration; patients who underwent percutaneous drainage of the abscess with either a single or double pigtail catheter. Patients with amebic abscesses and those with severe coagulopathy at admission were excluded. The doctor informed patients the possible risks and pain of the operation, and the patients chose to perform a single or double pigtail drainage catheters for treatment.

As per standard protocol, patients admitted with PLA were started on intravenous antibiotics. Treatment begun with parenteral third generation cephalosporins like cefoperazone sodium or with quinolones. In case the patient was not responding, the antibiotics were changed as per the sensitivity of the organism in the case of positive pus cultures or empirically to other drugs such as piperacillin tazobactam in the case of negative pus cultures. Intravenous antibiotics are given for 2–6 weeks depending on the response. In addition, the patients will be instructed to continue taking oral antibiotics for 2 weeks after discharge.

Two experienced physicians performed the operations. Percutaneous catheter drainage was done as follows: An 18-guage needle was inserted into the abscess cavity under ultrasound guidance and pus aspirated with a 20-ml syringe. A guidewire was inserted into the abscess cavity through the needle which was then removed. An or two 8F pigtail drainage catheters were then placed in the abscess cavity through the guidewire. The cavity was once flushed with 50–60 ml of normal saline every day thereafter.

**Figure 2:**
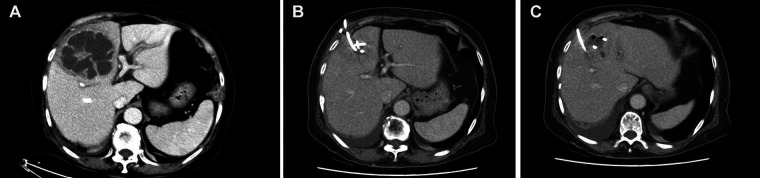
(**A–C**) two pigtail drainage catheters in the PLA and significant reduction of pyogenic liver abscess was observed on postoperative abdominal CT.

Routine monitoring of the patient included: clinical status especially the body temperature and other vital signs, amount of pus drained, white blood cell count, serum calcitonin, c-reactive protein, and liver and renal function tests. Pus aspirated was sent for culture and sensitivity. An abdominal CT scan or MRI was done as indicated for evaluation of changes in the size of the abscess cavity and the presence of blood or pus (Figures 2A–C). A review liver CT scan was done before removal of the catheter.

Data collected from the medical records included: demographic characteristics of patients, clinical features including presence of comorbidities, etiopathologic factors, laboratory and radiological findings, number, size and location of lesions, microbiological findings, details of treatment, treatment response, complications and mortality.

### Statistical analysis

Categorical data were expressed as numbers and percentages. Continuous data were summarized as mean + standard deviation. Continuous variables were compared using the Student's *t*-test or Wilcoxon test as applicable. Categorical data were compared using the chi-square test or Fisher's exact test. Logistic regression analysis was used for analysis of risk factors for septic shock. Statistical analysis was done using the IBM SPSS Statistics for Windows, version 23 (IBM Corp., Armonk, NY, United States) A *P*-value <0.05 was taken as statistically significant.

## Results

A total of 97 patients with PLA > 5 cm were treated with percutaneous drainage between May 2013 and May 2021. The baseline characteristics of patients who were treated with DP and single pigtail (SP) catheter drainage are summarized in Table 1. A majority of the patients in both groups were male. The right lobe of the liver was the most common location of the abscess. The two groups were comparable with respect to demographic characteristics, comorbid conditions and location and size of the liver abscess.

**Table 1:** Baseline characteristics of patients in the two groups.

	Double pigtail catheter drainage (*n* = 34)	Single pigtail catheter drainage (*n* = 63)	*P-*value
Age mean ± SD (years)	58.94 ± 14.33	59.27 ± 14.87	0.904
Gender			
Male *n* (%)	22 (65%)	39 (62%)	0.482
Female *n* (%)	12 (35%)	24 (38%)	
Location of abscess			
Left lobe *n* (%)	10 (29%)	15 (24%)	0.792
Right lobe *n* (%)	22 (65%)	45 (71%)	0.794
Both lobes *n* (%)	2 (6%)	3 (5%)	0.685
Diameter of abscess (mm)	79.7 ± 19.5	81.4 ± 24.4	0.107
Containing gas *n* (%)	7 (21%)	11 (17%)	0.315
Concomitant disease			
Diabetes mellitus	14 (41%)	25 (40%)	0.528
Hypertension	9 (26%)	16 (25%)	0.546
Cholelithiasis	5 (15%)	11 (18%)	0.483
Malignancy	4 (12%)	7 (11%)	0.584
History of abdominal surgery	4 (12%)	9 (14%)	0.495

Table 2 summarizes the clinical symptoms, laboratory tests, and complications of patients in the two groups. Fever and abdominal pain were the most common presenting symptoms of patients in both groups. Invasive liver abscess syndrome was seen in four patients. There was no difference between the two groups with respect to symptoms, laboratory findings or complications.

**Table 2:** Clinical symptoms, laboratory tests, and complications.

	Double pigtail catheter drainage (*n* = 34)	Single pigtail catheter drainage (*n* = 63)	*P-*value
Symptoms			
Fever *n* (%)	31 (91%)	58 (92%)	0.579
Abdominal pain *n* (%)	24 (71%)	39 (62%)	0.504
Generalized weakness *n* (%)	21 (62%)	37 (59%)	0.472
Cough *n* (%)	9 (6%)	21 (33%)	0.323
Nausea and vomiting *n* (%)	11 (32%)	19 (30%)	0.499
Laboratory results (mean ± SD)			
White blood cell count (×10^9^/L)	15.31 ± 5.22	13.84 ± 4.53	0.158
Platelet count (×10^9^/L)	26.91 ± 4.55	23.32 ± 5.83	0.659
C-reactive protein(mg/L)	134.19 ± 78.68	132.61 ± 69.26	0.709
Fibrinogen (mg/dl)	6.84 ± 1.58	6.42 ± 1.28	0.054
Alanine aminotransferase (U/L)	70.02 ± 46.23	74.52 ± 56.34	0.881
Total bilirubin (µmol/L)	25.83 ± 16.42	23.09 ± 14.58	0.447
Serum albumin (g/L)	27.09 ± 3.33	28.24 ± 4.48	0.112
Complications			
Pulmonary infection *n* (%)	13 (38%)	30 (48%)	0.273
Pleural effusion *n* (%)	25 (74%)	47 (75%)	0.546
Septic shock *n* (%)	4 (12%)	11 (17%)	0.335
ICU admission *n* (%)	1 (3%)	3 (5%)	0.562
Invasive liver abscess syndrome			
Endophthalmitis *n* (%)	1 (3%)	1 (2%)	0.654
Pulmonary abscess *n* (%)	1 (3%)	0	0.171
Subphrenic abscess *n* (%)	0	1 (2%)	0.351

ICU, Intensive Care Unit.

The microbiological findings are shown in Table 3. Positive cultures (pus and/or blood culture) were seen in 83 of 97 patients (89.2%). Gram-negative bacilli (Klebsiella pneumoniae and Escherichia coli) were the most commonly isolated organisms (82%) with Klebsiella pneumoniae being the most common (76%).

**Table 3:** Microbiological findings.

	Number of patients (total *n* = 97	Percentage (%)
Pus culture positive	77	79.4%
Blood culture positive	24	25%
Pus + blood culture positive	18	18.6%
Organisms		
*Klebsiella pneumoniae*	63	76%
*Escherichia coli*	5	6%
*Streptococcus*	5	6%
*Enterococcus faecium*	3	3.6%
Other bacteria	4	4.8%
Fungus	3	3.6%

Table 4 summarizes the post-operative course in the two groups of patients. The postoperative hospital stay, the time for removal of the catheter and the abscess diameter at time of removal of catheter were significantly lower in the DP group compared with the SP group. The rate of reduction in c-reactive protein levels (measured over three days) was also more rapid in the DP group.

**Table 4:** Comparison of the postoperative conditions between the two groups.

	Double pigtail catheter drainage (*n* = 34)	Single pigtail catheter drainage (*n* = 63)	*P-*value
Postoperative hospital stay (days)	13.39 ± 4.21	15.67 ± 7.50	0.045
Rate of reduction of c-reactive protein over 3 days (mg/l/day)	26.61 ± 14.11	20.06 ± 11.74	0.025
Complications			
Abdominal infection *n* (%)	0	1 (2%)	0.351
Postoperative fever and chill *n* (%)	22 (65%)	35 (56%)	0.398
Bleeding *n* (%)	0	0	
Recurrence of abscess within 6 months *n* (%)	0	1 (2%)	0.351
Time for removal of catheter (days)	17.23 ± 3.70	24.11 ± 5.83	0.038
Diameter of abscess at time of removal of catheter (cm)	3.1 ± 0.7	3.7 ± 0.6	0.047
Death in hospital *n* (%)	1 (3%)	2 (3%)	0.720

The risk factors for progression to septic shock were studied by univariate regression. Presence of gas in the abscess cavity and diabetes mellitus were further studied by multivariate regression. Only diabetes mellitus was found to be a significant independent risk factor for septic shock. The results of the regression analysis are shown in Table 5.

**Table 5:** Risk factors for septic shock—univariate and multivariate analysis.

Variables	Univariate Analysis	*P*-value	Multivariate Analysis	*P*-value
	Odds Ratio (95% CI)		Odds Ratio (95% CI)	
Gas in abscess cavity	3.908 (1.227, 12.450)	0.021	2.877 (0.795, 10.407)	0.107
Diabetes mellitus	5.026 (1.468, 17.206)	0.010	5.203 (1.358, 19.935)	0.016
Gender	1.599 (0.527, 4.856)	0.407		
Age	1.013 (0.974, 1.053)	0.516		
C-reactive protein	1.044 (0.997, 10.12)	0.294		
Abscess size	0.999 (0.975, 1.024)	0.967		

## Discussion

In recent years, antibiotic therapy along with PCD, rather than surgical drainage, has become the standard of care for PLAs (13, 14). PCD achieves the same cure rate as surgical drainage, but with less trauma, lower complication rates, and shorter hospital stay. Intravenous antibiotics are also the mainstay of treatment and are given for 2–6 weeks depending on the response.

In our study, the basic characteristics of the patients in the two groups were comparable. The mean age of presentation in our patients was around 59 years and more males than females were affected. Also, the right lobe of the liver was the most commonly affected. This is consistent with the findings in other studies. The incidence rate in men has been reported to be about 1.7 times higher than that in women, and the abscess was found more commonly in the right lobe of the liver (15, 16). Chan KS reported that the patients older than 65 would prolonged length of hospitalisation stay (17).

The common comorbid conditions seen in patients of liver abscess are diabetes mellitus, hypertension, malignant tumors, biliary stones, history of abdominal surgery, liver cirrhosis, and alcoholism (18). About 40% of patients in our study had diabetes and this is consistent with previous studies (19). Diabetes can lead to liver injury, abnormal bile secretion, and an increased chance of portal vein infection. It can also cause systemic metabolic impairment and reduced immunity, and weaken the ability of the liver to remove bacteria, thereby facilitating colonization of bacteria in the liver, leading to liver abscesses (1, 20, 21).

Studies have shown a higher proportion of use of carbapenem antibiotics in patients with liver abscesses complicated by diabetes. Poor glycemic control is associated with a higher rate of complications: difficulty in infection control, recurrence of abscess; and even death. Fifteen of our 97 patients also had septic shock. Multivariate analysis showed diabetes mellitus to be an independent risk factor for progression to septic shock in our patients. These observations suggest that PLA patients with diabetes may require more aggressive antibiotic therapy with carbapenem combinations (22, 23). Several population-based studies have shown that diabetes is a significant risk factor for PLA morbidity and mortality (14). Diabetes has been reported as an independent risk factor for mortality within six months in patients with PLA (24).

About 92% of our patients presented with fever and 55% with abdominal pain. Therefore, a diagnosis of liver abscess should be considered in patients presenting with persistent high fever and right upper abdominal pain. A total of 18 patients in our study demonstrated gas within their liver abscesses(18.6%), the result is similar to Chan KS'report (25). 72 had pleural effusion, and four had the invasive liver abscess syndrome. Zhang Jia et al. (26) reported a higher risk of pleural effusion and sepsis. Studies have also reported that the size of the abscess positively correlates with the severity of the disease. Larger PLAs are more prone to complications such as invasive liver abscess syndrome, pleural effusion, ascites, and abscess rupture. Also, the larger the PLA is, the longer the duration of hospitalization and the in-hospital mortality, independent of other risk factors (10, 27).

Du Zhao-Qing et al. found that pus cultures are more likely to be positive than blood cultures (14). This is consistent with the present study. All patients included in this study had a pus culture done. A majority of the patients also had blood cultures performed. As a result, we had a high rate of culture positivity (89.2%). Klebsiella pneumoniae was the commonest organism isolated (76%). Cultures are essential in guiding antibiotic therapy especially in immunocompromised patients. For patients with PLA without bacterial culture results, It is justified to treat them with empirical antibiotics targeted to K. pneumoniae (28).

Most PLA patients benefit from PCD, And there are fewer complications and adverse events, But PCD failures still occur (29). One patient in this study developed an inflow of pus into the abdominal cavity, Caused abdominal infection and acute peritonitis, It is considered because the abscess is located on the surface of the liver. Therefore, For abscesses located on the surface of the liver, It is recommended to puncture through more normal liver tissue. Avoid to puncture on the surface of the liver abscess. Patients of 58% with high fever and chills after PCD in this study, It is associated with inflammatory mediators in pus and bacterial entry into the blood. Although patients with liver abscesses generally have abnormal coagulation function, Bleeding complications after PCD treatment of liver abscess were relatively rare, all <1% (30). Many patients in this study performed 2 PCD procedures, No patients developed liver bleeding.

There are few reports on the study of double catheter drainage of PLAs and none comparing double catheter with single catheter drainage in PLAs (31). Double catheter drainage probably facilitates rapid emptying of the abscess cavity particularly in patients with large multiloculated lesions where a second drainage tube can be positioned in another part of the abscess. Rapid unobstructed emptying of the pus probably reduces the high bacterial load and inflammation, thereby decreasing the duration of hospitalization (32). In our study we observed a decrease in the time to removal of catheter and the duration of hospitalization in patients who had DP drainage compared with those treated with SP drainage. Besides, in patients with DP drainage, the time for reduction in the diameter of the abscess to half was shorter, the rate of decrease in c-reactive protein levels was more rapid (suggesting faster resolution of inflammation) and the size of the abscess at the time of catheter was smaller.

There is no consensus on the optimum time for removal of the percutaneous catheter. We opted to remove the catheter when the drainage through the unobstructed catheter was nil. Premature removal of the drain is not recommended, Although some studies have reported that the removal of drainage tubes with less than 10 ml of pus per day can be achieved a 90% successful treatment rate for larger liver abscesses (33). Carrying a drainage catheter increases the discomfort, However, there is still no study to confirm the safety and effectiveness of the early removal of drainage pipes. In that study, The average time for removal the catheter was 22 days. When remove the catheter, The mean diameter of the liver abscess was approximately 3.5 cm in these cases. All our patients were followed up for a minimum period of six months after discharge from the hospital, Only 1 case of patients hospitalized due to liver abscess recurrence.

Our study has certain limitations. Firstly, the study design was retrospective. Secondly, the number of patients was small. Thirdly A randomized controlled trial of double vs. single catheter drainage with an adequate sample size is necessary to establish whether double catheter drainage is superior or not to single catheter drainage in large PLAs. In this study, The 8F pigtail drainage catheters were applied in all patients. It was reported that the 10–12F catheters did not increase the risk of bleeding (28). We will try to use it in the future.

## Conclusion

Double catheter drainage is a safe and effective technique for drainage of pus in multiloculated PLAs more than 5 cm in size. We found double catheter drainage to shorten hospitalization time, time to removal of catheter and time for reduction in the size of the abscess cavity. The rate of reduction in inflammatory parameters like c-reactive protein levels was also more rapid in these patients.

## Data Availability

The raw data supporting the conclusions of this article will be made available by the authors, without undue reservation.
